# UV light-based decontamination: an effective and fast way for disinfection of endoscopes in otorhinolaryngology?

**DOI:** 10.1007/s00405-020-05978-w

**Published:** 2020-05-01

**Authors:** Stefan A. Rudhart, Frank Günther, Laura Dapper, Kruthika Thangavelu, Francesca Gehrt, Petar Stankovic, Thomas Wilhelm, Thomas Guenzel, Boris A. Stuck, Stephan Hoch

**Affiliations:** 1 Department of Otolaryngology, Head and Neck Surgery, University Hospital Marburg, Philipps-Universität Marburg, Marburg, Germany; 2 Department of Medical Microbiology and Hygiene, University Hospital Marburg, Philipps-Universität Marburg, Marburg, Germany; 3 Department of Otolaryngology, Head/Neck and Facial Plastic Surgery, Sana Kliniken Leipziger Land, Borna, Germany; 4 Medical Faculty, Philipps University of Marburg, Marburg, Germany; 5 Department of Otolaranygology, Head and Neck Surgery, Borromaeus Hospital, Leer, Germany

**Keywords:** UV light, UV disinfection, Endoscopes, Reprocessing, Otorhinolaryngology, Disinfection

## Abstract

**Background:**

Reprocessing of endoscopes becomes increasingly complex, due to rising demands of hygiene. Established methods are often expensive/time-consuming. Recent studies suggest beneficial aspects of disinfection by UV light. In this study we analyzed the efficiency of UV light disinfection of rigid otorhinolaryngological endoscopes.

**Materials and methods:**

After mechanical pre-cleaning, the endoscopes were decontaminated for 25 s in the D25 using Impelux™ UV C light technology (UV Smart B.V., Delft, The Netherlands). First, the surface contact samples were taken from 50 used endoscopes to evaluate the bacterial load. Additionally, surface contact samples were taken from further 50 used endoscopes after reprocessing with the D25. Another 50 endoscopes were tested on protein residuals. Furthermore, the absolute effectiveness of the D25 was tested on 50 test bodies (RAMS) with a standardized contamination of 10^7^ colony-forming units (CFU) of *Enterococcus faecium*.

**Results:**

The used endoscopes showed a high bacterial contamination with an average value of 66.908 (± 239.215) CFU. After reprocessing, only a minimal contamination on 10% (*n* = 5) of the endoscopes with a mean value of 0.12 CFU (± 0.39) was found, resulting in a log-5 reduction in a clinical environment. The documented bacteria were components of the normal skin flora. All tested endoscopes were practically protein-free (< 1 μg). Furthermore, the average absolute germ reduction of the D25 was about 10^6^ CFU on the tested RAMS.

**Conclusion:**

The D25 UV light system seems to be an effective device for the reprocessing of rigid ORL endoscopes, and therefore, might be suitable for the usage in clinical practice on site.

## Introduction

Endoscopic examination of the upper airways plays an important role in the management of nearly all diseases in otorhinolaryngology (ORL). Especially, rigid endoscopes (REs) are often used for the ORL examination due to their wide variety of indications and long lifetime [1, 2]. In outpatient departments with a high patient flow, the same RE is used and reprocessed many times a day between examinations of different patients. Therefore, reprocessing of the endoscopes is highly important to prevent transmission events. It is known that the risk of a transmission by endoscopes is mostly related to an insufficient reprocessing process before reusage [3]. Nowadays, endoscopy-related infections are a worldwide threat for healthcare systems. Several outbreaks of healthcare-associated infections with highly resistant bacteria clearly demonstrated the problem of contaminated endoscopes in the past [4]. According to the Spaulding’s classification system of medical equipment, REs are classified as semicritical patient care devices [5, 6]. Therefore, at least a high-level disinfection is required for REs before reuse [7, 8]. Till now, a large variety of different high-level disinfection methods exists and no standardization has been implemented within the field of otolaryngology. However, high-level disinfection is becoming increasingly complex due to the rising demands of hygiene and the increasing number of multi-resistant microorganisms. Established high level methods are often expensive and/or time-consuming, which may be problematic for daily clinical use, especially in an ORL outpatient department with a high patient flow.

Against this background, time- and cost-effective as well as safe and consistent methods for disinfection of the endoscopes are needed. In the literature, there are consistent data about the benefits of surface disinfection by UV light, which seems to be a suitable method for surface disinfection in case of hospital-acquired germs or biofilm-associated contaminations [9, 10]. The benefits of UV light-based disinfection are known since a long time. In this context, Nils Ryberg Finsen was one of the first, who used UV light to treat bacterial infections. In 1903, Finsen even won the Noble Prize for Medicine for the successful treatment of skin tuberculosis by UV light [11, 12]. Some years later, in 1930, first UV lamps became commercially available and were commonly used since 1945. At that time, when disinfection agents were not commonly available, low energy UV lamps were used in continuous operation to prevent infections in hospitals [13]. Over the years, UV light technology has advanced continuously. Today, UV light is commonly used for drinking-water disinfection, with the advantage compared to chemical methods of not having an influence on toxicity, taste or smell of the water [14]. To the best of our knowledge, UV light-based reprocessing methods have not been analyzed for rigid medical endoscopes in ORL before. Thus, in the present study, we evaluated the efficiency of the UV light in the reprocessing of REs.

## Materials and methods

### Reprocessing of endoscopes

The study was performed at a tertiary university outpatient department. In this context, the entire spectrum of patients was endoscopically examined. This included both infectious and non-infectious patients with various diseases in the head and neck-area. A selection of the analyzed endoscopes, according to the indication for its use, was not performed in order to get a representative cross-section of used endoscopes at an ORL outpatient department. In addition, the potential of UV light should be examined under “real-life” conditions and not only for a subgroup of patients. The effectiveness of the D25 UV light system (UV Smart, Delft, The Netherlands) for disinfection was evaluated for non-channel 30 and 70° REs (KARL STORZ SE & Co. KG, Tuttlingen, Germany) with a stainless-steel surface. Reprocessing was performed by mechanically pre-cleaning the endoscope for 20 s by a standardized water-based tissue (Tristel Rinse Wipes, Tristel GmbH, Berlin, Germany). Next, the endoscope was placed and decontaminated for further 25 s in the D25 UV light system. Each of the used endoscopes was decontaminated separately. After the cleaning process, the residual contamination was analyzed. Finally, to not interfere with the patient safety, each endoscope was reprocessed, analogous to the standard protocol of our clinic, by a washer–disinfector WD425E (Belimed, Zug, Switzerland).

### Microbiological examination/ protein testing of endoscopes

In the first step, surface contact samples on trypticase soy agar (Merck Millipore, Darmstadt, Germany) were taken from 50 endoscopes immediately after clinical use (rhinoscopy/laryngoscopy) without reprocessing in order to evaluate the mean bacterial contamination on the endoscopes. In the second step, 50 additional surface contact samples were taken from the used endoscopes after reprocessing, including pre-cleaning and disinfection by the D25 UV system. Microbiological samples were taken from the complete length of the shaft of the endoscope by rolling it over the agar plate. The quantification and identification of bacteria on the surface contact samples was performed after incubation at 37 °C overnight. Identification of bacteria was done by matrix-assisted laser desorption ionization time-of-flight mass spectrometry (Bruker Daltonik, Bremen, Germany).

Next, 50 endoscopes were tested after pre-cleaning and UV disinfection on protein residues as a marker for prion and viral contamination by Medi-Check™ (Hygiena Medisafe GmbH, Wentorf, Germany) test kit. After an incubation period of approx. 15 min at 55 °C, the Medi-Check™ test kit showed a change in color, with a range from 0 μg (light green) to 50 μg (dark gray/black), depending on the amount of protein residuals.

### Testing of absolute CFU reduction on RAMS test bodies

**Fig. 1:**
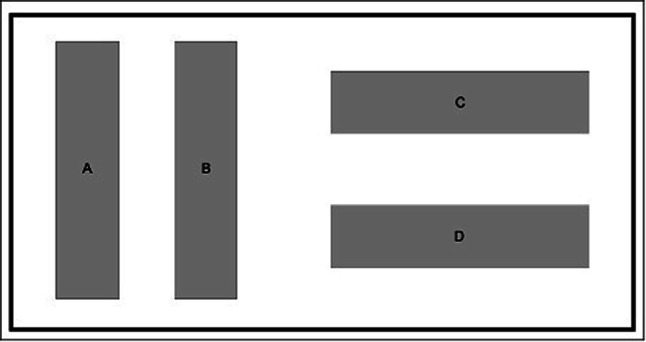
Arrangement of the RAMS test bodies in the D25 for testing the absolute bacterial reduction

For testing the potential of the D25 on absolute CFU reduction, 10-cm RAMS test bodies with a stainless-steel surface and a standardized *Enterococcus faecium* contamination of approximately 1×10^7^ CFU were used in the present study. Altogether, 51 test bodies were tested, while three were positive samples. The constituents of the RAMS test bodies (bovine albumin, mucein, and corn starch) represent an organic contamination and are usually used to test cleaning systems for the reprocessing of components of the highest category (critical) in Spaudings classification system [5]. Therefore, the testing conditions we used are compliable with the highest cleaning standards in medicine. In this part of the study, four RAMS test bodies were parallelly cleaned in each disinfection cycle. After each disinfection process, parts of the D25 facing the test bodies were cleaned with mikrozid^®^ sensitive wipes (Schülke & Mayr GmbH, Norderstedt, Deutschland). In each test cycle, two test bodies were placed longitudinally and two further ones transversely to the light sources in the UV box. The transversal positions were marked as position A and B while the longitudinal ones were marked as position C and D (Fig. 1). The contaminated RAMS test bodies underwent the same procedure of pre-cleaning and reprocessing as mentioned above for endoscopes.

### The D25 UV light system

**Fig. 2:**
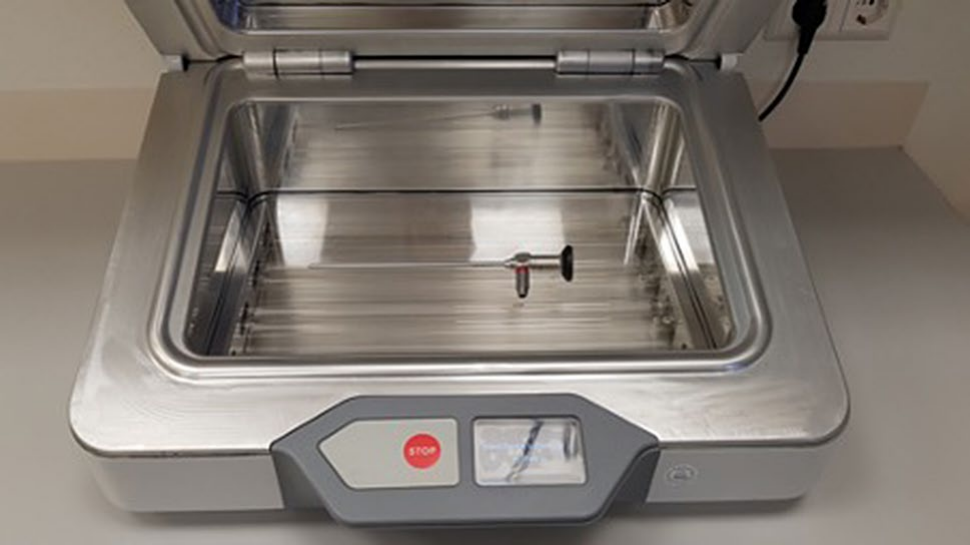
Arrangement of a rigid endoscope inside the D25

**Fig. 3:**
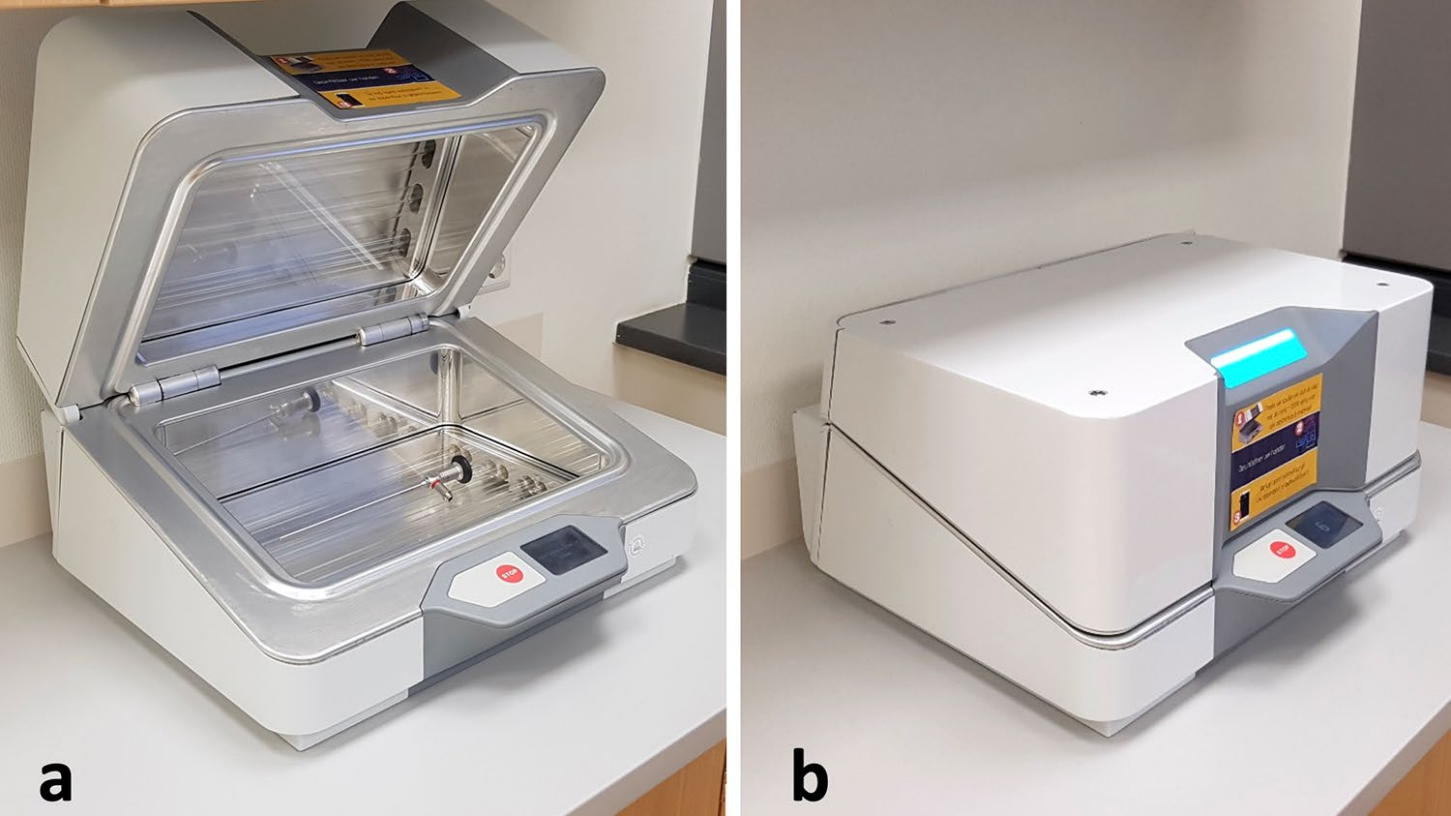
Open (**a**), and sealed (**b**) D25 UV light system for disinfecting an endoscope

**Fig. 4:**
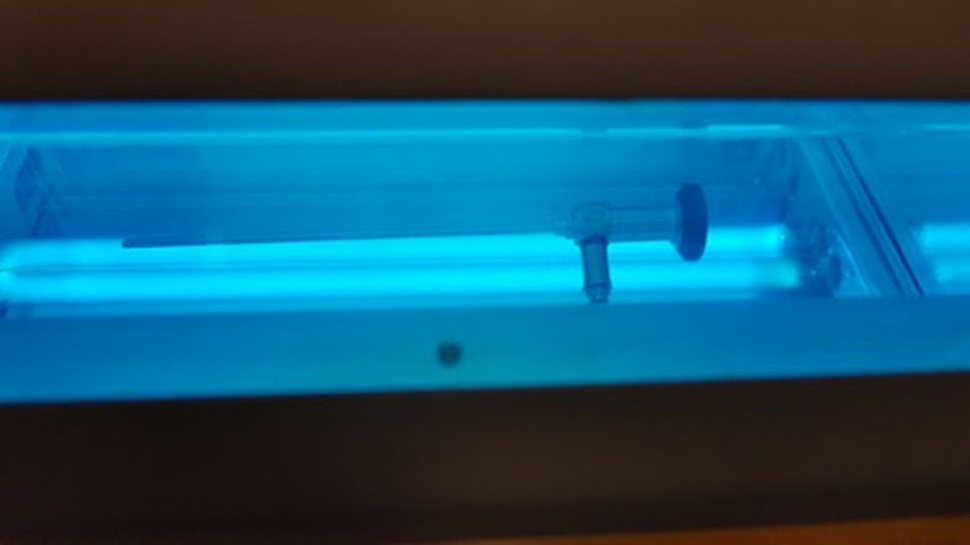
View inside the D25 during disinfection process

The UV Smart D25 disinfects medical devices physically in 25 s by using UV-C light. Except for pre-cleaning, no chemicals or liquids are needed for this process. To fit in the chamber, the medical devices should not exceed a maximum size of 150 mm height; 225 mm depth, and 380 mm width (Fig. 2). For operators’ and patients’ safety, the disinfection chamber is sealed while the lamps are applicating the UV-C light. Therefore, the UV-C light cannot escape the D25 (Figs. 3, 4). According to the results of the manufacturer’s internal investigations, the D25 achieves a bacterial reduction of 10^5^ in 25 s. Therefore, the manufacturer presets the device for a reprocessing time of 25 s.

The UV Smart Impelux™ technology in the D25 operates at a wavelength of 253.7 nm. During the cycle of 25 s, the D25 delivers a dose of 6872 µW/cm^2^. The Impelux™ technology causes a DNA, e.g., RNA damage in microorganisms on the surface of the inserted devices without affecting the devices surface itself. The germicidal effect of the Impelux™ technology and UV-C light will not penetrate dirt, debris, and grime. Therefore, it is required that all equipment and devices are visually clean before reprocessing in the D25.

### Statistics and ethical approval

Statistical analysis was performed by descriptive analysis with Excel 2019 (Microsoft Corporation, Redmond, WA, USA). The study was announced to the ethics committee of the Medical Faculty of the Philipps-Universität Marburg, and according to the statement of the committee, a formal approval was not necessary as no study-related measures were applied to subjects or patients.

## Results

**Fig. 5:**
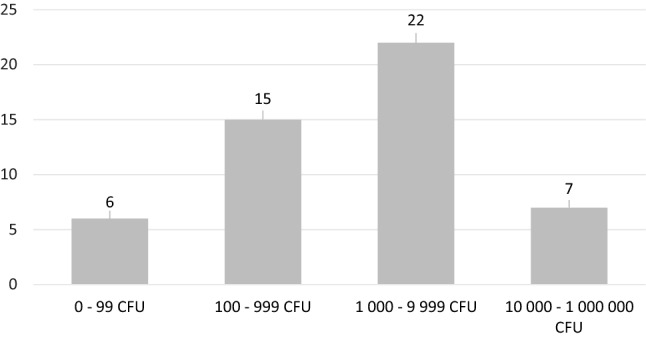
Number of the endoscopes depending on the amount of CFU found on their surface after clinical use without disinfection

Directly after clinical use without reprocessing, the 50 endoscopes showed a high bacterial contamination with a mean value of 66,908 CFU (± 239.215; 2–1,000,000 CFU) (Fig. 5). The bacterial contamination consisted of various bacteria, including normal skin flora, e.g. *Viridans streptococci* as well as pathogenic bacteria, such as *Pseudomonas aeruginosa*. The exact bacterial cultures found on the agar-plates and the number of endoscopes they were found on are listed in Table 1.

**Table 1:** Identified bacteria and the absolute number of endoscopes they were found on after clinical use without disinfection

Identified bacteria	Absolute number (%)
Coagulase-negative *Staphylococcus*	41 (82%)
*Micrococcus luteus*	17 (34%)
*Neisseria* species	13 (26%)
*Viridans streptococci*	11 (22%)
*Staphylococcus aureus*	11 (22%)
*Bacillus* species	4 (8%)
*Corynebacterium* species	3 (6%)
*Pseudomonas aeruginosa*	3 (6%)
*Haemophilus* influenzae	1 (2%)
*Escherichia coli*	1 (2%)
*Proteus* species	1 (2%)
*Enterobacter *cloacae	1 (2%)
*Enterococcus* species	1 (2%)
*Haemolytic streptococci*	1 (2%)

**Fig. 6:**
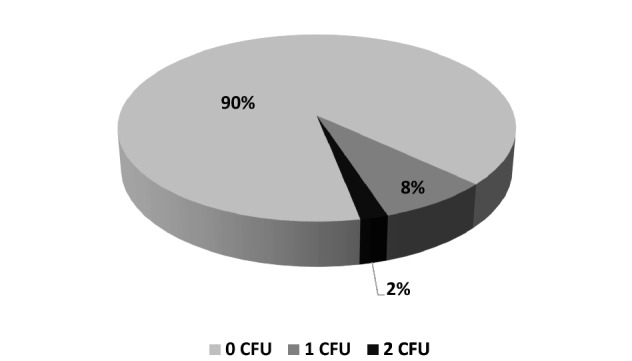
Percentage of endoscopes depending on the amount of CFU found on their surface after reprocessing

After the reprocessing, only a minimal contamination on 10% (*n* = 5) of the endoscopes with 1 CFU in 4 cases and 2 CFU in 1 case and thus a mean value of 0.12 CFU (± 0.39) was found. The documented bacteria after disinfection were attributed to the normal skin flora (coagulase-negative *Staphylococcus* and *Micrococcus luteus*). The remaining 90% (*n* = 45) of the samples showed no further bacterial contamination (0 CFU) (Fig. 6).

After pre-cleaning and reprocessing with the D25, all of the 50 tested endoscopes were practically protein-free (< 1 μg). Altogether, on 42 endoscopes, no residual contamination (0 μg) by proteins was found, while 8 endoscopes were minimally contaminated (< 1 μg) after reprocessing.

In the context of the test bodies reprocessing, the absolute bacterial contamination decreased significantly from 10^7^ CFU to an average value of 1.5 × 10^1^ CFU (± 60.88; 0–400 CFU) after disinfection. Altogether, 42 of the test bodies were completely free of bacteria, while 6 test bodies showed an average residual contamination of 2.9 × 10^1^ CFU (± 82.66; 20–400 CFU). The test bodies arranged longitudinally to the UV lamps (positions C and D) were completely free from bacteria, except one test body in position C, which was still contaminated with 6×10^1^ CFU. The test bodies arranged transversally to the UV lamps (positions A and B) were contaminated in 3 cases in position A and in 2 cases in position B after reprocessing (A: 2 × 10^1^, 6 × 10^1^, and 7 × 10^1^ CFU; B: 4 × 10^2^ and 1 × 10^2^ CFU).

## Discussion

Facing the danger of endoscopy-related cross-contamination and several healthcare-associated infections with highly resistant bacteria [4], UV light surface disinfection might play an important role in the future. Especially because, it is not influenced by resistance mechanisms or the biofilm of bacteria, and therefore efficient even against problematic multi-resistant pathogens [9, 10, 15]. Further, the UV light is effective for Gram-positive and -negative bacteria, as well as bacterial and fungal spores [16]. The UV radiation is absorbed by the DNA, e.g., RNA of the cells. As a result, thymine dimers are formed and DNA, e.g. RNA damage prevents gene expression and DNA, e.g. RNA replication [17]. This effect leads to apoptosis in the irradiated organisms. However, due to the physical damage of DNA, e.g. RNA, UV light also has a carcinogenic effect on irradiated cells. Hence, the usage of UV light requires an efficient isolation of humans, especially to prevent skin cancer [18]. Therefore, UV-based disinfection systems require a shielding against UV light at the time of application. In the case of the D25, the shielding against UV light is realized by its box-based design. However, due to its physical characteristics, the UV light does not penetrate most solid substances or unclear fluids. Therefore, pre-cleaning of the objects to be treated seems necessary to remove gross contamination. In the case of endoscopes used in ORL, secretion of the upper aerodigestive tract, solid nasal mucus or blood should be removed from the surface of the endoscopes before the application of UV light. Otherwise, disinfection of the surface would be inadequate. In the present study, we used a water-based tissue without any kind of disinfection agents, detergents or enzymatic components, in order to ensure that the disinfection effectiveness of the D25 is not affected by any pre-cleaning agents. However, water-based pre-cleaning is also a cost-effective, practical, and nearly-everywhere available procedure.

So far there is no sufficient data in the literature about the bacterial contamination of REs after clinical use on patients in ORL. Several factors have an influence on the contamination of the REs, including the time and amount of contact to the mucosa, the extent of bacterial colonization, and the varying infectivity between different patients. In the present study, we analyzed the bacterial contamination of REs under real clinical conditions. In this context, a high bacterial contamination was found on the non-disinfected endoscopes, with a high variety of the detected CFU, which might be explained by the influencing factors mentioned before. The germs found on the non-disinfected endoscopes in the present study are mainly in accordance with the normal mucosal flora of the upper aerodigestive tract [19]. However, even facultative pathogenic bacteria such as *P. aeruginosa*, which may cause severe, even life-threatening infections, were found on the surface of the endoscopes [20]. This stresses the necessity of a sufficient decontamination, to prevent a potential cross-contamination [4].

To the best of our knowledge, an UV applicating device for reprocessing of REs has not been explored so far. In the present study, we have demonstrated the potential of the D25 in efficiently reducing a high bacterial contamination on used endoscopes. After clinical usage on the patient, we found only a slight bacterial contamination with a maximum of 2 CFU in 10% of the analyzed endoscopes, which were decontaminated by pre-cleaning and UV light. The proven bacteria were related to the ordinary skin flora and no relevant protein residuals were detected. However, we tested the D25 under clinical conditions in our outpatient department to achieve more realistic results. Thus, the slightly residual bacterial and protein contamination may be influenced by handling the endoscopes and contact samples under unsterile conditions after disinfection. Therefore, further tests should be performed in a laboratory setting.

Testing the absolute bacterial reduction by using RAMS test bodies, an average germ load reduction of 10^6^ CFU (from 10^7^ CFU to 1.5 × 10^1^ CFU) could be observed in the present study. Therefore, the D25 fulfilled the requirements for semicritical devices as defined by the Commission on Hospital Hygiene and Infection Protection at the Robert Koch Institute (RKI) and the Federal Institute for Drugs and Medical Devices (BfArM) which require an average reduction of about 10^5^ CFU [21]. In the present study, we used RAMS test bodies, which are even harder to reprocess than the test bodies without bovine albumin, mucein, and corn starch as carrier substances. However, we found better results for test bodies placed in the longitudinal positions C or D than for the test bodies placed transverse in positions A or B in the device. These findings might be explained by the orientation of the test bodies to the lamps, which possibly influenced the disinfection power of the D25. However, endoscopes only fit longitudinal in the D25; therefore, this effect should be less relevant. Furthermore, the results might be influenced by the quality and intensity of pre-cleaning. Several studies proved the effectiveness of UV light-based disinfection methods in combination with mechanic cleaning, e.g., as a pre-cleaning. The results of these studies are controversially discussed, with a tendency to equal results of UV disinfection also without a mechanical pre-cleaning [22–25]. However, due to the physical mode of action of the UV-C light, it must be assumed that the protein concentration on the endoscopes is only reduced by the mechanical pre-cleaning.

In the literature, the distance of the UV light source to the object is discussed as a key issue for its disinfection efficiency [16]. In the D25 UV system, the distance from the lamps to the object is short, ranging from 2 to 18 cm. Further, the disinfection process of the D25 requires only 25 s, which is substantially less than other UV light-based disinfection systems that require minutes or even hours for surface disinfection [26, 27]. Another main concern in the context of disinfection is shadowing, which leads to less effective reprocessing [26]. The results of this study suggest that the arrangement of lamps and mirrors in the D25 with the Impelux™ UV light technology seems to cause a good light distribution and prevents shadowing inside the system. Hence, the disinfection process of the D25 seems to be highly effective.

In addition to its effectiveness, special attention must be paid to the efficiency of disinfecting endoscopes by using UV light, taking the costs for acquisition and maintenance of the D25 System, the required time and the associated human resource consumption into account. Assuming a reprocessing of 15,000 endoscopes per year over a time period of 5 years, the reprocessing cost per endoscope is calculated by the manufacturer to be about 0.17 euro with the D25. In our outpatient department, approx. 50–75 patients receive some kind of evaluation with one or more rigid endoscopes per day, which would correspond to 12,500–18,750 reprocessings per year. In comparison, the costs for disinfecting one endoscope amounts to about 4.50 euro for using chlorine dioxide wipes depending on the local delivery conditions and about 8.50 euro for a conventional automated cleaning by a washer–disinfector. Furthermore, conventional procedures may be time-expensive. Thus, the disinfection process with the D25 including precleaning takes about one minute. Common reprocessing methods usually take several minutes (chlorine dioxide wipes) to few hours (automated washer–disinfector). Although multiple endoscopes can be reprocessed in the automated washer–disinfector, the time and human resources for the manual transportation and handling of the endoscopes and the consecutive turnaround times need to be considered. Taking these aspects into account, reprocessing endoscopes using UV light seems to be an efficient method.

## Conclusion

The D25 UV light system seems to be an effective device for the disinfection of rigid ORL endoscopes. Moreover, the D25 device is a small-sized, fast, and easy disinfection method, and therefore, might be suitable for the usage in clinical practice on site, especially in the case of a high patient flow and small treatment rooms. It does not require constant supervision by the operator because the D25 stops and opens automatically when a reprocessing cycle has been completed. The device does not require the sealing of air conditioning or heating vents or windows in the room it is placed in. However, it is important to emphasize that pre-cleaning of the endoscopes seems to be necessary to reduce gross contamination by nasal or pharyngeal secretion, solid nasal mucus or blood on the endoscopes. Nevertheless, pre-cleaning by a simple water-based tissue seems to be adequate.

## References

[1] Armstrong M (2005). Office-based procedures in rhinosinusitis. Otolaryngol Clin North Am.

[2] Benninger MS (1997). Nasal endoscopy: its role in office diagnosis. Am J Rhinol.

[3] Seoane-Vazquez E, Rodriguez-Monguio R (2008). Endoscopy-related infection: relic of the past?. Curr Opin Infect Dis.

[4] Kenters N, Huijskens EG, Meier C, Voss A (2015). Infectious diseases linked to cross-contamination of flexible endoscopes. Endosc Int Open.

[5] Dales S, Mosbach EH, Lawrence CBS (1968). Chemical disinfection of medical and surgical materials. Disinfection, sterilization, and preservation.

[6] Ins E (2007). Ensuring the effective reprocessing of flexible endoscopes. Health Devices.

[7] Simmons BP (1983). CDC guidelines for the prevention and control of nosocomial infections guideline for hospital environmental control. Am J Infect Control.

[8] Muscarella LF (1996). High-level disinfection or “sterilization” of endoscopes?. Infect Control Hosp Epidemiol.

[9] Marra AR, Schweizer ML, Edmond MB (2018). No-touch disinfection methods to decrease multidrug-resistant organism infections: a systematic review and meta-analysis. Infect Control Hosp Epidemiol.

[10] Chen LH, Li Y, Qi Y, Wang SN, Gao CQ, Wu Y (2019). Evaluation of a pulsed xenon ultraviolet light device for reduction of pathogens with biofilm-forming ability and impact on environmental bioburden in clinical laboratories. Photodiagnosis Photodyn Ther.

[11] Finsen NR (1896). Om anvendelse i medicinen af koncentrerede kemiske lysstraaler.

[12] Nobel Lectures (1967). Physiology or Medicine 1901–1921.

[13] Fenton L, Moseley H (2014). UV emissions from low energy artificial light sources. Photodermatol Photoimmunol Photomed.

[14] Lyon BA, Milsk RY, DeAngelo AB, Simmons JE, Moyer MP, Weinberg HS (2014). Integrated chemical and toxicological investigation of UV chlorine/chloramine drinking water treatment. Environ Sci Technol.

[15] Rutala WA, Gergen MF, Weber DJ (2010). Room decontamination with UV radiation. Infect Control Hosp Epidemiol.

[16] Katara G, Hemvani N, Chitnis S, Chitnis V, Chitnis DS (2008). Surface disinfection by exposure to germicidal UV light. Indian J Med Microbiol.

[17] Wacker ADH, Weinblum D (1960). Strahlenchemische veränderung der bakterien-desoxyribonucleinsäure in vivo. Naturwissenschaften.

[18] Deshmukh J, Pofahl R, Haase I (2017). Epidermal Rac1 regulates the DNA damage response and protects from UV light-induced keratinocyte apoptosis and skin carcinogenesis. Cell Death Dis.

[19] Todar K (2006) Todar’s online textbook of bacteriology. University of Wisconsin-Madison, Department of Bacteriology Madison, USA. 10.1007/s00103-012-1548-6

[20] El Zowalaty ME, Gyetvai B (2016). Effectiveness of antipseudomonal antibiotics and mechanisms of multidrug resistance in pseudomonas aeruginosa. Pol J Microbiol.

[21] (KRINKO) CfHHaIP (2012) [Hygiene requirements for the reprocessing of medical devices. Recommendation of the Commission for Hospital Hygiene and Infection Prevention (KRINKO) at the Robert Koch Institute (RKI) and the Federal Institute for Drugs and Medical Devices (BfArM)]. Bundesgesundheitsblatt Gesundheitsforschung Gesundheitsschutz 55 (10):1244–1310. 10.1007/s00103-012-1548-6 10.1007/s00103-012-1548-6 23011095

[22] Green C, Pamplin JC, Chafin KN, Murray CK, Yun HC (2017). Pulsed-xenon ultraviolet light disinfection in a burn unit: impact on environmental bioburden, multidrug-resistant organism acquisition and healthcare associated infections. Burns.

[23] Vianna PG, Dale CR, Simmons S, Stibich M, Licitra CM (2016). Impact of pulsed xenon ultraviolet light on hospital-acquired infection rates in a community hospital. Am J Infect Control.

[24] Jinadatha C, Villamaria FC, Restrepo MI, Ganachari-Mallappa N, Liao IC, Stock EM, Copeland LA, Zeber JE (2015). Is the pulsed xenon ultraviolet light no-touch disinfection system effective on methicillin-resistant staphylococcus aureus in the absence of manual cleaning?. Am J Infect Control.

[25] Jinadatha C, Villamaria FC, Ganachari-Mallappa N, Brown DS, Liao IC, Stock EM, Copeland LA, Zeber JE (2015). Can pulsed xenon ultraviolet light systems disinfect aerobic bacteria in the absence of manual disinfection?. Am J Infect Control.

[26] Boyce JM, Havill NL, Moore BA (2011). Terminal decontamination of patient rooms using an automated mobile UV light unit. Infect Control Hosp Epidemiol.

[27] Haas JP, Menz J, Dusza S, Montecalvo MA (2014). Implementation and impact of ultraviolet environmental disinfection in an acute care setting. Am J Infect Control.

